# The role of natural selection in human evolution – insights from Latin America

**DOI:** 10.1590/1678-4685-GMB-2016-0020

**Published:** 2016-08-04

**Authors:** Francisco M. Salzano

**Affiliations:** 1 Departamento de Genética, Instituto de Biociências, Universidade Federal do Rio Grande do Sul (UFRGS), Porto Alegre, RS, Brazil

**Keywords:** natural selection, human evolution, population genetics, human adaptation, history of genetics

## Abstract

A brief introduction considering Darwin's work, the evolutionary synthesis, and the scientific biological field around the 1970s and subsequently, with the molecular revolution, was followed by selected examples of recent investigations dealing with the selection-drift controversy. The studies surveyed included the comparison between essential genes in humans and mice, selection in Africa and Europe, and the possible reasons why females in humans remain healthy and productive after menopause, in contrast with what happens in the great apes. At the end, selected examples of investigations performed in Latin America, related to the action of selection for muscle performance, acetylation of xenobiotics, high altitude and tropical forest adaptations were considered. Despite dissenting views, the influence of positive selection in a considerable portion of the human genome cannot presently be dismissed.

## History

### Darwin and the Evolutionary Synthesis

Everything started with the flash of genius of Charles Darwin (1807-1882), which occurred in 1842, followed by a careful review of the available information up to 1859, when he published his seminal work, "The Origin of Species" (Darwin, 1859). In this book he maintained that all the phenomenal variability observed in the biological world could only be understood under the principle of natural selection, which he defined as "The preservation of favorable individual differences and variations, and the destruction of those which are injurious".

He hesitated in extending this principle to the human species, and only in 1871, 12 years later, he published "The Descent of Man" (Darwin, 1871) in which he applied the concepts of natural selection, and especially sexual selection, to the evolution of our species. A brief outline of this book is provided in Table 1. As indicated, the work is divided in three parts; the first reviews the evidence that humans have derived from previous forms; the second is dedicated to sexual selection; and the third to sexual selection in its relation to humans, and to basic conclusions.

**Table 1:** The main contents of Darwin's "The Descent of Man".

**I. The descent or origin of humans** I.1 Proofs of descent of human from some inferior forms I.2 How humans developed from some inferior forms I.3 Comparison between the mental abilities of humans and of inferior animals I.4 Development of the intellectual and moral faculties during the primitive and civilized ages I.5 Human affinity and genealogy I.6 The human races **II. Sexual selection** II.1 Principles of sexual selection II.2 Sexual secondary characteristics in the inferior classes of the animal world II.3 Sexual secondary characteristics of insects II.4 Sexual secondary characteristics of fishes, amphibians, and reptiles II.5 Sexual secondary characteristics of birds II.6 Sexual secondary characteristics of mammals **III. Sexual selection in relation to humans and conclusion** III.1 Human sexual secondary characteristics III.2 Conclusion

Source: Darwin (1871).

What are these conclusions, and the added comments? He maintained that the whole body of evidence undeniably indicated that we had derived from an animal ancestor. In relation to objections of the church to this view, he asserted that faith in God had been considered the most completed distinction between humans and animals, but it would be impossible to maintain that this faith is innate or instinctive in humans; and that if it was considered non-religious to explain the human origin from a distinct species by natural selection, then the same would apply for the explanation of the birth of individuals through the laws of normal reproduction.

Darwin made a clear distinction between natural and sexual selection. According to him, sexual selection depends of the success of some individuals in relation to others of the same sex regarding the species reproduction, while natural selection depends on the success of both sexes, at all ages, in relation to this process. More generally, he added that he would rather prefer to be considered a descendant of a non-human primate than to persons who torture the enemies, consider women as slaves, have no shamefacedness, and are tormented by an enormous amount of superstitions.

One of the weaknesses of Darwin's theory, which he himself recognized, was the ignorance at the time of the laws that determined the biological inheritance in living organisms. But the fundamentals of these laws were clearly delineated in 1866, seven years after the publication of "The Origin of Species", by Gregor Mendel (1822-1884). Mendel's work, however, was not recognized by his peers, and only 34 years later (1900) it was rediscovered and duly appreciated.

The fusion between Darwin's and Mendel's works occurred in two very fertile decades of the 20^th^ century, between 1930 and 1950, through the Synthetic Theory of Evolution. Elsewhere, in Chapter 1 of Herrera *et al*. (2016), I listed the 11 key books that provided the fundamentals of this theory. Parallel developments in paleoanthropology and in human population genetics (cf. Salzano, 1977), provided the framework to relate the principles of the synthesis to events which occurred with ourselves in the past.

### The picture around the 1970s

In 1870, Alfred Russel Wallace (1823-1913) who developed independently of Darwin the concept of the key role of natural selection in biological evolution, questioned its role in relation to our species. How could the conventional natural selection select the exceptional capacities developed by the human mind? The answer would be provided in the 20^th^ and 21^st^ centuries by a series of empirical data and mathematical models.

**Figure 1:**
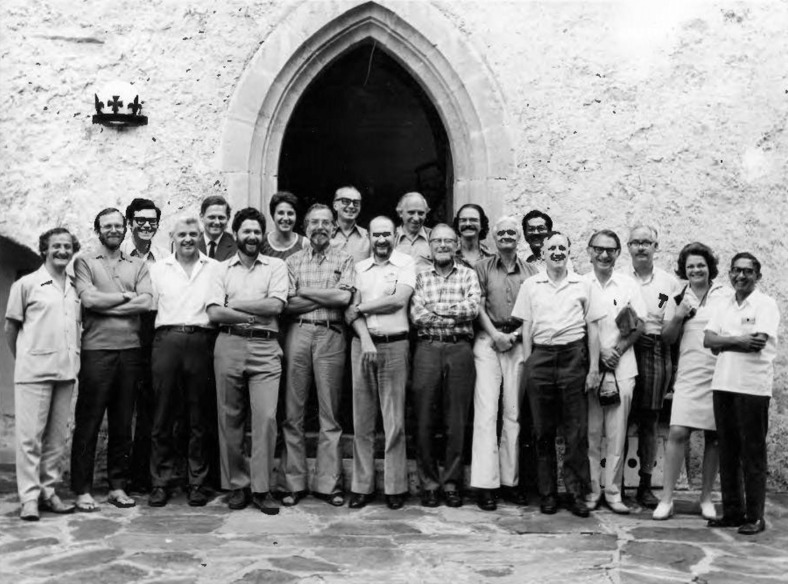
Group photograph of the participants of the 1974 Burg Wartenstein Symposium, The Role of Natural Selection in Human Evolution. First row, from left to right: 1. P.V. Tobias; 2. N.E. Morton; 3. W.M. Fitch; 4. J.S. Friedlander; 5. J. Huizinga; 6. J. Gomila; 7. J.V. Neel; 8. F. Vogel; 9. D.F. Roberts; 10. A.G. Motulsky; 11. T.E. Reed; 12. L. Osmundsen; 13. L.D. Sanghvi. Second row, also left to right: 14. F.J. Ayala; 15. B. Clarke; 16. P.A. Jacobs; 17. N. Freire-Maia; 18. H. Harris; 19. F.M. Salzano; and 20. S. Ohno.

At the end of the sixties Kimura (1968) and beginning of the seventies Kimura and Ohta (1971) argued that natural selection would not be important at the molecular level, and that at this level the most important factor would be random genetic drift of selectively neutral mutant alleles. Concerned with this and related work I decided to assemble a selected group of 19 prominent scholars of distinct disciplines to consider the different aspects of natural selection in relation to our species. The meeting occurred in 1974, in an ideal place for such gatherings, Burg Wartenstein, Austria, sponsored by by the Wenner-Gren Foundation for Anthropological Research, and was ably administered by Lita Osmundsen and her staff (Figure 1). Its proceedings were published as a book one year later (Salzano, 1975).

Table 2 lists the book's main contents and the corresponding contributors. It was divided in seven sections: (a) Molecular variation; (b) Hominid evolution; (c) Population structure; (d) Quantitative and qualitative traits distributions and the factors influencing them; (e) The nature of our genetic load; (f) The role of culture; and (g) Synthesis. In the final chapter the state of the art in these different fields was briefly considered, the methods used surveyed, and suggestions for further work given. The conclusion related to M. Kimura and T. Ohta proposals was that although their theory has been of considerable heuristic value, its main arguments were considerably weakened after the tests to which they have been submitted.

**Table 2:** The main contents of the book "The Role of Natural Selection in Human Evolution", published in 1975.

**I. Molecular variation: findings and interpretation** I.1 Scientific hypotheses, natural selection and the neutrality theory of protein evolution, by F.J. Ayala I.2 Evolutionary rates in proteins and the cost of natural selection: implications for neutral mutations, by W.M. Fitch I.3 Rates of biochemical and chromosomal changes in the vertebrates, by S. Ohno I.4 Enzyme polymorphisms and the neutral hypothesis of molecular evolution, by H. Harris **II. Hominid evolution** II.1 Long or short hominid phylogenies? Paleontological and molecular evidences, by P.V. Tobias **III. Population structure** III.1 Models of population structure and reality, by J.S. Friedlander II.2 Kinship, fitness, and evolution, by N.E. Morton II.3 Fertility differentials and their significance for human evolution, by J. Gomila II.4 Frequency-dependant and density-dependent natural selection, by B. Clarke **IV. Causal factors in the distribution of quantitative and qualitative traits** IV.1 Natural selection and human morphology, by T. Bielicki IV.2 Interpopulation variability in polymorphic systems, by F.M. Salzano IV.3 Selection and the blood group polymorphisms, by T.E. Reed IV.4 ABO blood groups, the HLA system and disease, by F. Vogel IV.5 G6PD and abnormal hemoglobin polymorphisms – evidence regarding malarial selection, by A.G. Motulsky **V. The nature of our genetic load** V.1 Adaptation and genetic load, by N. Freire-Maia V.2 The genetic consequences of inbreeding, by L.D. Sanghvi V.3 The load due to chromosome abnormalities in man, by P.A. Jacobs **VI. The role of culture** VI.1 The study of "natural" selection in man: last chance, by J.V. Neel VI.2 Fertility, mortality and culture: the changing pattern of natural selection, by D.F. Roberts VI.3 Cultural and biological adaptation in man, by J. Huizinga **VII. An attempt at a synthesis** VII.1 Some key problems in the study of natural selection in man, by F.M. Salzano and all contributors

Source: Salzano (1975).

### The molecular revolution and the opportunity for in-depth analyses

Methods for studying population variability had progressed at a fast rate, especially in the last years. In the 19^th^ century morphological traits were investigated using qualitative, obtained by visual inspection, or quantitative methods, through the use of anthropometric measurements. During the 20^th^ century immunological and biochemical techniques were employed to study variation at the protein level, but towards the end of this century and the present one it became possible to study the genetic material directly through, among others, the use of the polymerase chain reaction (PCR) technique and by increasing sophisticated sequencing machines. Parallel developments in bioinformatics and the implementation of the Internet made possible increased interaction between research teams, with the formation of consortia for the study of specific problems at the world level; and the establishment of data banks, with the possibility of analysis of a huge amount of data. The opportunity was open, therefore, to investigate problems that were defying solution, such as the selection-drift controversy.

In what follows I will present some selected examples of work performed by different groups of researchers, giving emphasis, at the end, of studies performed by our research team. Before that, however, I would call attention to a recent review I made of the selection or drift debate in Chapter 2 of Herrera *et al*. (2016). This review considered the history, methods, and analyses related to this subject. First, the way to detect selection and mutation effects at the intra and interspecific levels was examined; second, seven specific methods that could be used for detection of selection based on DNA sequences and single-nucleotide polymorphisms (SNPs) were listed, indicating their utility and limitations; and third five of them directly applicable to the human genome (high proportion of function-altering mutations; reduction in genetic diversity; high-frequency derived alleles; interpopulation differences; and long haplotypes) were considered. This evaluation was made based on the work of Nielsen (2005), Sabetti *et al*. (2006), Harris (2008), Akey (2009), Zhong *et al*. (2010), and Peter *et al*. (2012).

The review ended by listing selected examples of studies that could be classified as follows: (a) six emphasizing the role of mutation, demography, and drift for the explanation of human genomic variability; (b) five that considered the role of negative selection on this variability; and (c) fifteen emphasizing the role of positive selection in the human genome. The conclusion was that the influence of positive selection in a considerable fraction of our genome cannot be dismissed. But dissent views continue to be presented. A highly selected list would include Lynch (2007), Koonin (2012) and Nei (2013).

## Selected examples of recent investigations

### What portion of our genome is essential for survival?

The most dramatic effect that can occur in the phenotypic expression of a gene is loss of essentiality. Genome-wide gene-deletion in the experiments with information about the fitness of each deletion strain had been performed in the bacterium *Escherichia coli* and in the yeast *Saccharomyces cerevisae*. They therefore can be used as sources of comparison with data on humans. Liao and Zhang (2008), however, preferred such a comparison with a more closely related species, and devised a study summarized in Table 3. Surprisingly, of a total of 120 human-mouse orthologs 27 (22%) that were essential for humans were verified as nonessential for mice; and an assessment of protein sequence rates of evolution disclosed an accelerated rate in these genes, driven by positive selection in the human branch.

**Table 3:** Example of an organismal approach to the assessment of human essential genes.

**I. Method** I.1 Initial set of genes considered: 1,716 disease genes listed in OMIM^1^ I.2 Fraction with mouse orthologs that have been experimentally deleted and the results cataloged in MGI^2^ I.3 Final list, after pruning, of genes leading to death before puberty or infertility: 120 **II. Results** II.1 A total of 27 (22%) of the 120 mouse orthologs of human essential genes are nonessential II.2 Gene expression evolution is not the cause of the changes in gene essentiality in the two species II.3 Accelerated protein sequence evolution driven by positive selection was associated with changes in gene essentiality in at least an appreciable fraction of the 27 genes mentioned in II.1

1 OMIM: Online Mendelian Inheritance in Man database.

2 MGI: Mouse Genome Informatics database.

Source: Liao and Zhang (2008).

An alternative approach that would make human comparisons with microorganisms feasible was followed by Wang *et al*. (2015). Their investigation is outlined in Table 4. In this case 10% of 18,166 genes targeted by their approach proved to be essential for optimal cell proliferation. Evolutionary analyses indicated that these essential genes were more broadly retained across species, showed higher levels of conservation between closely related species, and presented fewer inactivating polymorphisms in humans as compared with corresponding nonessential genes. In this case, therefore, the action of negative selection was clearly established.

**Table 4:** Example of a cellular approach to the assessment of human essential genes.

**I. Methods** I.1 Use of the bacterial clustered regularly interspaced short palindromic repeats (CRISPR) system to serum for genes required for proliferation and survival in the near-haploid human KBM7 chronic myelogenous leukemia (CML) cell line I.2 Independently, generation at random through retroviral gene-trap mutagenesis, selection for a phenotype, and monitoring by sequencing the viral integration sites to pinpoint the causal genes I.3 For both methods, computation of a score for each gene that reflects the fitness cost imposed by the inactivation of the gene I.4 Comparison with functional profiling experiments conducted in the yeast *Saccharomyces cerevisae* I.5 Comparison of two CML and two Burkitt's lymphoma cell lines **II. Results** II.1 Of the 18,166 genes targeted by the library, 1878 (10%) scored as essential for optimal proliferation II.2 Results with the second method (item I.2) provided essentially the same picture II.3 Essential genes were more broadly retained across species, showed higher levels of conservation between closely related species, and contained fewer inactivating polymorphisms in humans, than their dispensable counterparts II.4 Essential genes also tended to have higher expression and encoded proteins that engage in more protein-protein interactions II.5 Differences in essential genes in the four cell lines studied might represent attractive targets for antineoplastic therapies

Source: Wang *et al*. (2015).

### The African Genome Variation Project and selection in Africa

The African Genome Variation Project comprises a network of collaborative research teams from Africa, Europe and USA, and a recent publication describing its findings is summarized in Table 5. A large population sample of 1,481 persons from 18 ethno-linguistic groups was studied using deep genotyping and whole-genome methods. A large amount of single nucleotide polymorphisms (29.8 million!) was detected, leading to important new knowledge on the populations of the continent. Outlying high differentiation alleles distinguishing Africans from Europeans were employed to determine the presence of positive selection, and frequencies from geographical areas with high prevalences of infectious diseases compared with those from regions free of them. These approaches resulted in the identification of novel gene regions implicated in genetic susceptibilities to malaria, Lassa fever, trypanosomasis, and trachoma, as well as others related to osmoregulation, and essential and secondary hypertension. Obviously, these findings have important medical implications, emphasizing how genetic/genomic information can contribute to this area of knowledge.

**Table 5:** Main findings of the African Genome Project.

**I. Subjects and methods** I.1 A total of 1,481 individuals from 18 ethno-linguistic groups living in sub-Saharan Africa were studied I.2 DNAs were investigated with the Human Omni 2.5M genotyping array and whole-genome sequences from 320 individuals were obtained **II. Results** II.1 Not less than 29.8 million single-nucleotide polymorphisms (SNPs) from Ethiopian, Zulu, and Bagandan whole-genome sequences were found II.2 A substantial proportion of unshared (11%-23%) and novel (16%-24%) variants were observed, with the highest proportion found among Ethiopian populations II.3 Highly differentiated SNPs between European and African populations, as well as among African populations, were examined for indications of selection in response to local adaptive factors II.4 Enrichment of loci known to be under positive selection was observed among the most differentiated sites II.5 Novel gene regions related to malaria, Lassa fever, trypanosomiasis, and trachoma susceptibilities, osmoregulation, and essential and secondary hypertension were identified

Source: Gurdasani *et al*. (2015).

### Natural selection in Europe

The modern sequencing techniques, coupled with enhanced methods of ancient DNA capture and study, are opening perspectives undreamed even a few years ago. The work by Mathieson *et al*. (2015) can be chosen to demonstrate the power of such approach, and a summary of their findings is displayed in Table 6. The DNA of a total of 230 ancient individuals from Eurasia dated to 6.5-0.3 kya who lived in different areas of Western Eurasia were studied and compared with genome-wide information from extant persons. Clear signs of selection involving diet, pigmentation, immunity, and height were obtained, placing in a firm basis previous reports suggesting the importance of this factor on these phenotypic traits.

**Table 6:** Genome-wide patterns of selection in Europe.

**I. Subjects and methods** I.1 A total of 230 ancient individuals from West Eurasia dated to 6.5-0.3 kya^1^ was studied I.2 In solution hybridization with synthesized oligonucleotide probes was performed; the targeted sites included nearly all SNPs on the Affymetrix Human Origins and Illumina 610-Quad arrays, and specifically 47,384 SNPs with evidence of functional importance I.3 Genetic affinities were tested considering 1,055,209 autosomal SNPs when analyzing the 230 ancient individuals alone, or 592,169 SNPs when co-analyzing them with 2,345 present-day persons genotyped on the Human Origins array **II. Results** II.1 Significant signals of selection on major genes **Table:** Genes Potential function *MCM6*, LCT Lactase persistence *SLC45A2* Skin pigmentation MHC region Immunity *FADS1*, *FADS2* Fatty acid metabolism *TLR1*, *TLR6*, *TLR10* Immunity *ATXN2*, *SH2B3* Unknown *DHCR7*, *NADSYN1* Vitamin D metabolism *GRM5* Skin pigmentation *SLC22A4* Ergothioneine transport *ZKSCAN3*, *ZSCAN31* Autophagy, Lung function *Chr13 rs 1979866* Unknown *HERC2*, *OCA2* Eye color II.2 In addition, evidence of selection on height was also obtained

1 kya=kilo (one thousand) years ago.

Source: Mathieson *et al*. (2015).

### Aging and selection

Senescence can be defined as the age-related deterioration of organismal function and fitness. Must all organisms age? The answer seems to be yes, since the overwhelming majority of metazoans show clear aging signs, and even unicellular organisms present this phenomenon through asymmetrical cell division (the two products of a cell division are not identical, and those that inherit the younger regions live longer).

The classical explanation for the existence of aging is antagonistic pleiotropy. Any favorable mutation acting early in life would be positively selected even if it increases the risk of death or infirmity later in life.

As far as humans are concerned, women's fertility ends at about the same age that fertility ends in other females of the great apes; but while our closest living relative became decrepit and die soon, female humans can remain healthy and productive well past menopause. Why? The answer may be cooperative breeding – the grandmother, older siblings, and other members of the extended family can help mothers to raise their children in a healthy way.

On the other hand, increased maternal age at reproduction is often associated with decreased offspring performance in numerous species of plants and animals, including humans. Moorad and Nussey (2016) considered this problem by the development of a model which tested for both direct and indirect genetic effects, and concluded that fertility and maternal effect senescence can experience different patterns of age-specific selection in relation to neonatal survival. Their main conclusion was that wherever fertility senescence occurs, selection for maternal effects will tend to decline more rapidly with age than selection for fertility.

So far, so good, but what if elders who contribute to cooperative breeding show a deterioration of their cognitive capacities? Schwarz *et al*. (2016) investigated the contribution of an immunoregulatory receptor, CD33, to a uniquely human post-reproductive disease, Alzheimer's dementia. Surprisingly, even though selection at advanced ages is expected to be weak, a CD33 allele protective against this disease is derived and unique to humans. They also found several other examples of derived alleles at other human loci that protect against age-related cognitive deterioration. Therefore, selection for inclusive fitness may be strong enough to favor these types of alleles, maximizing the contributions of post-reproductive individuals to the fitness of younger kin.

## Examples from Latin America

### Genetic variability influencing muscle performance

High quality muscular constitution is of course of prime importance in humans. This type of ability may have been especially significant during the time when our species had hunter-gathering as a way of subsistence. Both high speed (needed to flee from predators) and endurance running (providing persistence hunting and easier access to carcasses) would be important in the early days of our evolutionary history.

The human alpha-actinin skeletal muscle isoform 3 (*ACTN3*) gene is located in the long arm of chromosome 11, codifying a binding protein related to fast twitching the muscle fibers. Its lack leads to a slower, but more efficient aerobic pathway. A C >T transition at the ACTN3 557 residue converts arginine to a premature stop codon; this change is polymorphic (rs 1815739), and surveys conducted at different regions of the world have led to the data summarized in Table 7. The allele's frequencies vary all the way from a low 9% in Africa to a high 76% in Native Americans.

**Table 7:** Prevalences of ACTN3*R577X around the world.

Geographical populations	No. of individuals studied	ACTN3*R577X- frequencies (%)
Africa	794	9.3
Middle East	356	39.2
Europe	445	44.3
Central and South Asia	199	50.2
East Asia	581	47.7
Oceania	35	49.5
Americas	394	76.4

Source: Amorim *et al*. (2015a).

Previous studies had suggested that selection could be involved in the determination of these frequencies, and Amorim *et al*. (2015a) tested this suggestion by: (a) applying the Relative Extended Haplotype Homozygosity (REHH) test, which indicates the amount of linkage disequilibrium present in an area associated with a given allele that is being subjected to change. Higher values of this index suggest selective sweeps; (b) estimating the age of the mutation event; and (c) devising five demographic models that would be associated to the Native American/Asian split.

The allele's emergence was dated to 61.4 kya, in the range of time calculated for the first human migrations out of Africa, and the pattern of frequencies showed a general trend of increase with distance from this continent. The REHH values obtained from Asians and Europeans indicated the presence of an adaptive sweep, but the numbers from the Native American populations did not reach statistically significant results. The latter could be due to the peculiar population structure of Native Americans, which favors high interpopulation variability, in part due to random drift. But drift alone cannot explain the allele distribution found; rather, it points to a combination of drift and selection, with possible changes in selective pressures over time and space, and the loss or fixation of the beneficial allele in certain groups due to drift.

### Genetic variability and modes of subsistence

In the course of human evolution we have adopted several modes of subsistence. In the beginning we had to rely on hunter-gathering; subsequently, with the domestication process, we became agriculturalists and/or pastoralists; while with further developmental socioeconomic processes human groups became urban, with an industrialized way of subsistence. Each of these modal types, on the other hand, was associated with different population structures. Hunter-gatherers had a fission-fusion type of structure, characterized by nomadism and frequent splits and merging of small bands; agriculturalists or pastoralists with an island-type structure, characterized by small or medium-sized communities separated by large distances; while in urban and industrialized societies the populations could be described as following an isolation by distance model (Neel and Salzano, 1967; Salzano, 1972; Salzano and Callegari-Jacques, 1988). The patterns of genetic variability would be expected to markedly differ according to these types of structure, with obvious evolutionary implications.

A test to this expectation was recently made by our research group. It was based on the examination of the haplotype population distribution in an interesting genetic system, N-acetyltransferase 2 (NAT2). The corresponding alleles are located on chromosome 8p22, in a gene with two exons, only one of which presents variation. NAT2 is an enzyme responsible for catalyzing the N- or O-acetylation of aromatic and heterocyclic amines and hydrazines present in a wide range of xenobiotics and other substances, including medicines and food. In particular, it was found that the enzyme is responsible for the genetic metabolic response to isoniazid (an antituberculosis drug) and many other commonly prescribed drugs, such as sulfamethoxazole, used in the treatment of Acquired Immune Deficiency Syndrome (AIDS).

NAT2 polymorphisms lead to distinguishable haplotypes, associated with high or reduced enzymatic activities, which were correlated with fast (ancestral type) or slow (derived) acetylator/metabolizer phenotypes. The slow condition can lead to collateral effects of disease treatment, as was well demonstrated for isoniazid. Based on a worldwide analysis of this polymorphism, Sabbagh *et al*. (2011) suggested that populations practicing farming and herding would have higher prevalences of the derived (slow) acetylation phenotypes. Since not much was known at the time in relation to this polymorphism in Latin American populations, Bisso-Machado *et al*. (2015) decided to investigate it there, furnishing also new data to test the hypothesis.

The results obtained are summarized in Table 8. Four haplotypes, one the ancestral, associated with the rapid phenotype (*4) and the remaining three with the derived, slow condition, are responsible for 90% to 100% of the genetic constitution of the Latin American and Siberian subjects studied. As a comparison, note the much larger variability observed in Africa (these four haplotypes are responsible for only 64% of their genomes). Eskimo and Native American hunter-gatherers do not differ markedly in relation to these haplotypes, but the same is not true in relation to the three sets of other Native Americans or the two Mestizo population samples studied. No significant differences were found between hunter-gatherers and agriculturalists, but the number of hunter-gatherer individuals tested was not large (166+76=242). The marked interpopulation variability found among hunter-gatherers (0.11; P <0.001), not found in the agriculturalist set, could also have contributed to the lack of significance between hunter-gatherers/agriculturalists. The interpopulation variability difference observed, however, is compatible with that expected according to the genetic models outlined above.

**Table 8:** NAT2 haplotype frequencies and mode of subsistence in populations around the world.

Population	Mode of subsistence	No. studied	NAT2 haplotypes (%)^1^				
			*4	*5B	*6A	*7B	Others
**Native Americans**							
Mesoamericans	Agriculturalists	16	35	25	0	44	6
Amazonian and Central Brazil	Hunter-gatherers	166	31	36	5	25	3
Southern	Agriculturalists	68	44	10	6	38	2
Chaco	Agriculturalists	44	77	14	0	9	0
**Mestizo/Amerindian**							
Mexicans	Urban	68	25	10	16	46	3
Andeans	Agriculturalists	134	36	25	16	13	10
**Siberians**							
Eskimo	Hunter-gatherers	76	26	35	25	12	2
**Africa**	Several	2,100	10	30	22	2	36
**Europe**	Several	9,848	22	40	27	2	9
**Asia**	Several	8,500	48	11	23	10	8

1 The phenotype associated to *4 is rapid; for the three others, slow.

Sources: For the Native Americans, Mestizo/Amerindians and Siberians: Bisso-Machado *et al*. (2015); for the continental sets used as comparison, Sabbagh *et al*. (2011).

### Adaptation to high altitudes

There is a voluminous literature related to human high altitude adaptation. A classical interdisciplinary investigation was performed by Paul T. Baker and associates in the 1960s (Brooke-Thomas, 2009). More recent studies in Amerindians were summarized by Frisancho (2013). It is suffice to mention here that such adaptation involves an intricate set of both environmental and genetic variables. Here I will only mention a recent study provided by our group (Jacovas *et al*., 2015) in relation to a set of genetic markers that already were shown to play an important role in blastocyst implantation and recurrent pregnancy loss, investigated in lowland South America as opposed to high-altitude Andean populations.

The systems studied, related to the Tumor Protein 53 (TP53) pathway, have been extensively investigated in relation to disease associations, but much less studied for its population variability and related evolutionary significance. Our research was concentrated in five single nucleotide polymorphisms of genes from this pathway: (a) TP53 gene and associated p53 protein, which act by activating or repressing a large number of target genes related to metabolism and apoptosis, rs 1042522; (b) E3 ubiquitin-protein ligase, MDM2, which mediates the activity of TP53; rs 2279744; (c) Protein MDM4, a negative regulator of p53, rs 1563828; (d) Ubiquitin-specific-processing protease 7, USP7, which deubiquitylates p53, protecting it from proteasome degradation, rs 1529916); and (e) Leukemia Inhibitory Factor, LIF, a cytokine expressed in various cell types, favoring blastocyst implantation, rs 929271.

While variation in the TP53 polymorphism itself furnished non-significant results, *USP7-G*, *LIF-T*, and *MDM2-T* as well as their interactions, showed significant evidence that they were selected in relation to the harsh environmental variables related to high altitudes in the Andes, and probably connected to the well-known reproductive adaptation of Andean women to these conditions. A secondary effect could be related to the reported lower cancer incidences observed in Andean high altitude groups.

### Convergent genome-wide signals of adaptation to tropical forests

Tropical forests, characterized by high temperatures and rainfall, dense vegetation, as well as an enormous pathogen diversity, are, undoutedly, a very harsh environment for humans; and it is unclear whether we would ever have subsisted there without depending on external sources, such as agriculture or exchange with neighboring populations. They provide, therefore, an ideal model for the investigaton of natural selection, and an attempt in this direction was made by Amorim *et al*. (2015b).

The investigation included not less than 660,918 SNPs, tested using Illumina chips, studied in Mandenka and Yoruba (grouped as West Africa), African Biaka and Mbuti pygmies (chosen to provide what are considered classical cases of tropical forest adaptation), Suruí, Karitiana, Pima and Zapotec Native American populations. They were compared in different sets, according to whether they inhabited (Biaka, Mbuti) or not (Mandenka, Yoruba) in Africa or the Americas (respectively Karitiana, Suruí; Pima and Zapotec) tropical forests.

After a series of sophisticated analyses, seven regions (clusters), located in five different chromosomes, were detected as showing clear indications of positive selection related to tropical forest adaptations. The main genes related to them are listed in Table 9. They are involved in diet, muscle development, immunity, detoxification, regulation of body temperature, cell proliferation, sperm function, and vascular and calcium metabolism variables. Clusters 1 and 2 show evidences of this positive selection in the Americas only; Clusters 3 and 4, in Africa only; and the remaining three in both continents, therefore providing examples of convergent evolution. Other genomic regions were found to be associated with body height both in Africa and the Americas, furnishing an additional example of convergent evolution.

**Table 9:** Genes with signals of positive selection suggesting human adaptations to tropical forests in Africa and the Americas.

Gene	Chromosome and cluster^1^	Biological function
*SCP2*	1,1	Cholesterol trafficking and metabolism
*CWH43*	4,3	Lipid metabolism
*DCUN1D4*	4,4	Muscle development
*LRRC66*	4,4	Muscle development
*SGCB*	4,4	Muscle development
*SPATA1*	4,4	Muscle development
*C5orf34*	5,5	Unknown
*CLL28*	5,5	Immunity to viral infections
*NNT*	5,5	Free-radical detoxification
*HSF2*	6,6	Regulation of body temperature
*PKIB*	6,6	Cell proliferation
*SERINC1*	6,6	Unknown
*FKBP6*	7,7	Sperm function
*NSUN5*	7,7	Vascular system, calcium metabolism
*TRIM50*	7,7	Vascular system, calcium metabolism

1 Clusters 1 and 2 show indications of positive selection in the Americas; Clusters 4 and 5 positive selection in Africa; Clusters 6-7, convergent evolution in Africa and the Americas.

Source: Amorim *et al*. (2015b).

## Conclusion

This review included investigations performed outside and inside Latin America involving genes that are essential for survival in humans and mice, traits of medical importance in Africa, ancient and modern DNA studies in Europe, human aging, muscle performance, acetylation of xenobiotics, and high altitude and tropical forest adaptations. As can be inferred by the wide variety of subjects considered, it is now impossible to deny the importance of natural selection in shaping a considerable portion of the human genome. But there is much work still to be done. Especially important would be methodological advances for the analysis of different types of selection regimes, such as selection on standing genetic variation and the dynamics of polygenic systems.
